# Hypothesis: improving literacy about health workforce will improve rural health workforce recruitment, retention and capability

**DOI:** 10.1186/s12960-019-0442-9

**Published:** 2019-12-30

**Authors:** Alexandra Martiniuk, Richard Colbran, Robyn Ramsden, Dave Karlson, Emer O’Callaghan, Estrella Lowe, Michael Edwards, Sharif Bagnulo, Imogene Rothnie, Laura Hardaker, Bernadette Gotch, Arna Wotherspoon

**Affiliations:** 1 The University of Sydney, Edward Ford Building A27, Sydney, NSW 2006 Australia; 2 The George Institute for Global Health, City Road, Sydney, NSW 2006 Australia; 3 NSW Rural Doctors Network, PO Box 1111 Mascot, Sydney, NSW 1460 Australia; 4 Deakin University, 221 Burwood Highway, Burwood, VIC 3125 Australia

**Keywords:** Health, Workforce, Literacy, Rural, Recruitment, Retention, Capability

## Abstract

**Background:**

One of the key barriers to health in rural areas is health workforce. Poor understanding and communication about health workforce across all stakeholder groups (including the broad community) is very common and can negatively affect the health workforce, recruitment, experiences and outcomes.

**Hypothesis:**

In this paper, we propose the concept of literacy about health workforce. We propose this as a specific, actionable extension of the existing and well accepted health literacy concept. We hypothesise that improving literacy about health workforce will improve, in particular, rural health workforce recruitment, retention and capability.

**Implications of the hypothesis:**

We propose that literacy about health workforce is important for all members of the health and broader system (e.g. local GP, mayor, workforce agency, health manager, Aboriginal health worker, carers, community health facilitators, patients, schools, local businesses, cultural and recreation groups) because we hypothesise their literacy about health workforce affects their capacity to make informed decisions and take action to manage their health workforce needs in direct synchrony with the community’s health needs. We hypothesise that improving literacy about health workforce will improve the effectiveness and efficiency of attracting, recruiting, training, and retaining a high quality, capable, health workforce, and further, will support the development and acceptance of innovative solutions to health workforce crises such as new models of care. This hypothesis is action orientated, is testable and includes the consideration of methods to engage and improve literacy of those within and external to the health workforce.

## Introduction

In this paper, we propose to extend the concept of health workforce literacy to literacy *about* health workforce. We propose this as an extension of the existing and well accepted health literacy concept. We hypothesise that improving literacy *about* health workforce will improve, in particular, rural health workforce recruitment, retention and capability.

Our hypothesis is that by improving literacy about health workforce, measureable benefits with respect to rural health workforce would follow (e.g. improvements in rural health workforce recruitment, sustainability, capability and quality of life). This is a hypothesis that is yet untested. The testing of this hypothesis will be quantitative. The counter hypothesis is that improving literacy about health workforce makes no measureable improvement on any workforce or health outcomes.

## What is health literacy?

Health literacy refers to the ability of a person to access, understand and use information to make decisions and take action about health and healthcare [1]. Individuals (communities) and organisations can be health literate. At the organisational level, health literacy refers to the way services are provided and the processes in place to assist people to access, understand and use information. At the individual level, health literacy is important because poorer health literacy can lead to poorer health, increased hospitalisations, reduced self-management of health conditions, medical mistakes and greater health expenses [2]. Poor health literacy reduces people’s ability to access health care (for instance knowing when, who, where) and reduces the ability to share relevant information and collaborate in care. People with poor health literacy have less knowledge about “illnesses and injuries, treatment options, what causes ill-health and the importance of healthy lifestyles.” [3] They are less likely to use programmes that keep them healthy or enable them to take action early. By comparison, high levels of health literacy improve health-related behaviours, appropriate use of health services and navigation of the health system [4]. Health literacy is an accepted concept and has a growing evidence base. Searching “health literacy” in Medline (May 102 019) returns 8612 articles (5147 of these in the last 5 years).

## Health workforce needs

It is well known that a high-quality, sustained, health workforce contributes to a healthy population. In Australia and globally, new vertical initiatives are implemented (e.g. end rheumatic heart disease [RHD] in rural Australia, or HIV care in Africa) yet rarely are the workforce implications taken into account [5, 6]. Attention to health workforce is crucial if we want to support a healthy nation/world.

A report from the Australian Productivity Commission recognises that Australia is now predominantly a service economy and says that health care is a service as well as an economic driver. In the last decade, the Australian health workforce has grown from 1.1 million to 1.6 million people and now represents 13% of the total Australian workforce [7]. By comparison, nationally in Australia, work in retail, mining and manufacturing has stayed the same. Focus on the health workforce and improving its distribution, quality and capability will not only improve health, it will improve the cost effectiveness of health systems and boost the overall economy.

Approximately 7 million people live in regional, rural and remote areas of Australia. One of the key enablers and/or barriers to health in rural areas is health workforce.

## The idea of literacy *about* health workforce

We propose a focus on “literacy ABOUT health workforce” and propose a hypothesis related to this. While this may not be a brand new concept—it extends existing ideas of organisational, system and workforce literacy. Literacy ABOUT health workforce has not been defined, described, researched or tested for its utility in improving health workforce recruitment, retention and capability especially for rural communities. As well, while the newly summarised term “organisational health literacy” [8] appears to discuss a similar concept to “literacy ABOUT health workforce” the term “organisational health literacy” continues to refer to “organisations within the health system” and the concept we are proposing—refers to both organisations within the health system but, arguably more importantly, organisations outside of the health system.

We propose that literacy about health workforce is important for all members of the system (e.g. local GP, mayor, workforce agency, health manager, Aboriginal health worker, carers, community health facilitators and patients) because we hypothesise their literacy about health workforce affects their capacity to make informed decisions and take action to manage their health workforce needs in direct synchrony with the community’s health needs. Target audiences for this hypothesis include those named above as well as: administrators, policy makers, funding organisations including philanthropists, community leaders and community members themselves (patients). In terms of patients (community members), having greater literacy about health workforce might influence thinking about what they need (i.e. that a GP, or physiotherapist or nurse can meet a particular health need).

Literacy about health workforce could be said to be the degree to which individuals (and organisations) have the capacity to obtain, process and understand health workforce information and services needed to make appropriate health workforce decisions (Martiniuk modified from definition of health literacy by Parker et al) [9]. We hypothesise that improving literacy about health workforce will improve the effectiveness and efficiency of attracting, recruiting, training and retaining a high-quality, capable, health workforce, and further, will support the development and acceptance of new models of care. This could be tested.

Health workforce literacy extends from the existing health literacy concept whereby the people involved are patients, health professionals and health care organisations. Literacy about health workforce goes on to include all sectors. The information and actions in health workforce literacy are not about a particular health condition or how to navigate a hospital’s hallways but instead aim to improve literacy (knowledge) about the health system, the population health needs, how to recruit a new health professional, evidence about why health professionals stay in their roles, training pathways, registration and supervision requirements and so on. It is a literacy which remains in health organisations but also steps outside of hospitals and clinics and is needed (but lacking) in town halls, by public servants, community leaders, practice owners, recruiting agencies and so on. It includes and goes beyond existing workforce literacy concepts of increasing patients’ and health workforce’s understanding of the roles of the different professions, organisations and systems [10, 11].

## Existing knowledge leading to our proposed hypothesis

The ideas in this paper arose within the New South Wales Rural Doctors Network (RDN) Australia. Like many working in human resources for health, RDN has spent over 30 years building and supporting the NSW Rural Primary Health Workforce. RDN is an independent non-governmental organisation (NGO) working in partnership with all levels of government, health training and service organisations, communities and health professionals. RDN works with communities on locally driven solutions and advances the capacity and capability of rural primary healthcare professionals and organisations. Our untested hypothesis arose from data gathered by RDN including New South Wales (NSW) and National Minimum Data Set Reports on the rural primary health workforce [12].

RDN staff have noted that “often, the real problem is not the problem everyone is complaining about because they don’t understand how A connects to B and connects to C.” Several examples have been provided by senior RDN staff where strong or weak health workforce literacy in a community impacted its ability to support and grow its rural primary health care workforce. For instance, this quotation is a good illustration of what can happen when people have increased literacy regarding health workforce “Once you get an agreement in that community about what is required, they just take off [solving the issue successfully].” There are real barriers in not only the understanding, but updating resources, informing new practitioners and the community about what is available through the health workforce and what health needs are actually being met. All of the senior staff at RDN agree this concept has value; “we do this all the time, but we do it on an individual basis as the conversations unfold - we should be thinking of this from a system point of view”.

## Hypothesis

A “health workforce-literate community” recognises that poor understanding and communication about health workforce is very common and can negatively affect the health workforce, recruitment, experiences and outcomes. It also understands that making it easier for people to navigate, understand and use information as well as community capital and resources related to rural health workforce training, recruitment, retention and capability needs to be a priority (Martiniuk modified from the NSW Clinical Excellence Commission statement on health literacy) [13].

We hypothesise that if a “community” is engaged and understands the health workforce sector, the scope of health professional types and the sector’s complexities, they will be more understanding and capable in making informed decisions and acting on health workforce-related issues as well as supporting health workforce initiatives for their communities. Literacy about health workforce may help bring people “onto the same page” so to speak, and this may improve workforce capability through joint action across stakeholders. Ultimately, we hypothesise that if a community has high health workforce literacy then we will see improvements in attracting, recruiting and retaining a capable rural primary health workforce. By “communities” we include government staff, health administrators, key decision and policy makers, service organisations and civil society communities. By “primary care health workforce”, we include the broadest catchment: primary health workforce as medical, allied health, nursing, midwifery, Aboriginal health workforce, pathology, imaging, pharmacy, carers and community health facilitators.

The health workforce “system” is very complex and most communities (e.g. mayor, existing health professionals, local individuals) have difficulty understanding and using currently available information to support attraction, recruitment and retention of health professionals. As well, health professionals are no longer standalone entities (the solo GP of days past), but are now more often part of a primary healthcare professionals team. The difficulties understanding health workforce are understandable given rural health workforce recruitment often occurs through the existing health workforce, local government councils and civil society groups who are not trained in health workforce matters [14]. For individuals living in rural communities, this often means they lack the equitable access to quality health care because there is either insufficient capacity or insufficient capability of the local health workforce to meet their health needs. This may be in part due to the insufficient capability (in part potentially due to weak literacy about health workforce) in the community to attain and sustain the appropriate health workforce.

We believe a concerted effort should be made to improve health workforce literacy across government, private sector, not-for profit organisations, communities and individuals. The concept of literacy about rural health workforce can go beyond working with each rural community but the concept can also underpin or infuse how organisations work together in rural health. Supporting the broader community to become more literate about rural health workforce can also help inform policy development and improve decision making and programme design—impacting health workforce at the community level. Increased literacy about health workforce may encourage each of the necessary, interacting groups in health workforce to work proactively, and at a systems level, to support existing health professionals and plan succession for key roles well in advance of gaps in the workforce emerging.

Increasing literacy about health workforce may help communities manage their own health workforce, empower communities to be partners in their own health workforce issues, improve access to and understanding of health workforce information and develop strategies and initiatives to help navigate the workforce “system” (Martiniuk modified from health literacy sentences in the NSW State Health Plan: Toward 2021) [15]. A strong literacy about health workforce may improve community and health workforce capability, as well as improve the effectiveness and efficiency in securing and sustaining the required health workforce in rural communities. When we refer to workforce capability, we are hypothesising that improving literacy about health workforce may improve individuals’ and groups’ ability to innovate, be adaptable and be open to change and contribute to finding solutions. Could health workforce literacy contribute to the mechanisms for innovating, acting and utilising different options and opportunities in meeting the health needs of communities?

Improved literacy about health workforce may support knowledge continuity in the rural health sector. RDN and others have observed the detrimental impact of high turnover in the rural workforce, not only in the health service delivery workforce but also in executive and senior management roles across the rural health sector. Increased literacy about health workforce may assist in improving workforce knowledge of staff in rural health which may contribute to maintaining a level of consistency and balance across the sector and assist with briefing people new into organisations and roles about health workforce generally as well as locally. Crotty, Henderson and Fuller [16] found that health services in rural communities rely on informal networks from existing personal relationships and shared knowledge to improve service delivery and collaboration. Potentially increasing literacy about workforce will catalyse these processes and reduce the burden high workforce turnover causes. Without continuity there can be major disruption to projects and programmes and collaborative work plans for communities, regions and jurisdictions. The historical aspects of workforce policy are critical to understand in designing workforce programmes and maximising the levers to benefit rural health. We also hypothesise that increasing literacy about health workforce may better support the recent wave toward commissioning health services. Currently, rural communities suffer from issues due to commissioning by external service providers who lack local knowledge and on the other hand local service providers find themselves unable to compete for tenders [17].

In addition to supporting workforce recruitment and retention, we hypothesise that strong health workforce literacy is a key ingredient to health workforce innovation, including identifying and utilising substitute workforces and models needed to respond to population health needs—especially in remote and Indigenous communities. Strong health workforce literacy we hypothesise is also essential for high-quality service planning and has significant benefits in developing efficacious and cost-effective services.

We hypothesise that health workforce literacy is a key element of integrated and multi-disciplinary care. Teams operate better when everyone understands their role and scope of practice, as well as those of their team mates. This, as well as the integration of intersecting models of care (e.g. telehealth, specialist outreach) is better achieved with strong literacy about health workforce.

Improved literacy about health workforce in communities may reduce the burden on workforce agencies where the need for workforce support is growing rapidly and unsustainably. This is plausible since we know that self-management is improved with improved health literacy. For governments, improving literacy about health workforce may ultimately reduce costs through shared understanding, easing processes, improving planning and reducing workforce crises (e.g. sudden health workforce vacancies causing loss of critical services such as maternity care) to name some possible benefits.

Just as health literacy reduces health inequities so too literacy about health workforce could reduce inequities observed in rural communities’ ability to secure and retain high-quality health workforce compared to urban settings. We also know that low health literacy leads to poorer outcomes and under-utilisation of existing services. This may be the same for literacy about health workforce.

## Where to next?

This paper explores the possibility of the extension of health literacy to include literacy about health workforce. There is potential value to improving literacy about health workforce. Next, there is a need to better understand what literacy about health workforce means. This might mean greater exploration into modifying existing health literacy domains for a workforce lens, e.g. (a) scientific literacy (what are the health needs of the community, what workforce is required, including understanding of health workforce teams, e.g. need for anaesthetist if full maternity services are required or need for supervision); (b) civic literacy (e.g. understanding the policies, organisations, processes in health workforce attraction, retention); (c) cultural literacy (understanding the current who’s who/who does what in health workforce in the community, region, state) [18].

Further ideas from health literacy can be used for health workforce literacy—for instance supporting communities and organisations to make greater use of “plain language” documentation, using lists, flow-charts and simpler explanations to better educate others about health workforce processes. We need to better understand what literacy about health workforce is lacking, at what level, and produce “curriculum/education” to fill these voids. This will include ensuring easy access to workforce information and navigation assistance. Modifying existing frameworks, theories and tools from health literacy will be a start but to develop the concept of literacy about health workforce and to make it useful in practice we will likely need to go far beyond modifications into gathering new evidence and ultimately learn more about the impact and value of improving health workforce literacy. This will be the testing phase of the hypothesis. New evidence for health literacy [19] as well as literacy about health workforce, we propose, is particularly required for rural health settings.

While still under development, we have begun to explore approaches to testing the hypothesis that improving literacy about health workforce will improve the recruitment, retention and capability of the rural health workforce. Some potential approaches which we may use to test this hypothesis include (a) a case study methodology using existing routinely collected data, (b) qualitative in-depth interviews with a broad range of stakeholders in the rural primary health system and (c) development and testing of a “programme” to improve literacy about rural health workforce; for instance in a cluster randomised control trial (RCT) design, or stepped-wedge RCT design, to assess whether improving literacy about the rural health workforce can improve upon the outcomes (such as recruitment, retention and capability of the rural health workforce).

The advent of national action plans for health literacy (in Australia, USA and Scotland amongst others) have supported the patient-centred care and shared decision making movements and may be empowering the community to contribute to health system reforms [20]. Expanding health literacy to include literacy about health workforce may facilitate new thinking and action for health workforce in challenging settings (such as rural health and low- and middle-income countries). We believe that strengthening literacy about health workforce assists in implementing national and state action plans for rural health; aligning with local state policies in NSW such as Building a Sustainable Health Workforce for Rural NSW, The NSW Health Professionals Workforce Plan 2012-2022, The NSW Rural Health Plan amongst others.

## Abbreviations

GP: General practitioner
HIV: Human immunodeficiency virus
NGO: Non-governmental organisation
NSW: New South Wales
RCT: Randomised control trial
RDN: Rural Doctors Network
RHD: Rheumatic heart disease
